# Gas discrimination by simultaneous sound velocity and attenuation measurements using uncoated capacitive micromachined ultrasonic transducers

**DOI:** 10.1038/s41598-021-04689-4

**Published:** 2022-01-14

**Authors:** Luis Iglesias Hernandez, Priyadarshini Shanmugam, Jean-François Michaud, Daniel Alquier, Dominique Certon, Isabelle Dufour

**Affiliations:** 1 Laboratoire IMS UMR-CNRS 5218, University of Bordeaux, 351 cours de la libération, 33405 Talence Cedex, France; 2 GREMAN, UMR-CNRS 7347, University of Tours, 16 rue Pierre et Marie Curie, BP 7155, 37071 Tours Cedex 2, France

**Keywords:** Chemistry, Nanoscience and technology, Physics

## Abstract

Chemically functionalized or coated sensors are by far the most employed solution in gas sensing. However, their poor long term stability represents a concern in applications dealing with hazardous gases. Uncoated sensors are durable but their selectivity is poor or non-existent. In this study, multi-parametric discrimination is used as an alternative to selectivity for uncoated capacitive micromachined ultrasonic transducers (CMUTs). This paper shows how measuring simultaneously the attenuation coefficient and the time of flight under different nitrogen mixtures allows to identify hydrogen, carbon dioxide and methane from each other and determine their concentration along with identification of temperature and humidity drifts. Theoretical comparison and specific signal processing to deal with the issue of multiple reflections are also presented. Some potential applications are monitoring of refueling stations, vehicles and nuclear waste storage facilities.

## Introduction

Nowadays, most gas sensors present a chemical component such as a functionalized coating specifically engineered to target a particular analyte^1,2^. However, it is well known that such chemical coating presents long term stability issues^3^. This results in the need for recalibrating the sensor, typically every couple of months. For applications dealing with hazardous gases such as hydrogen, $\text {H}_{2}$, or methane, $\text {CH}_{4}$, this becomes dangerous for the operators. Alternatively, it is possible to compensate this drift digitally, for instance, with artificial intelligence^4^ or sophisticated models^5^. Nevertheless, this naturally increases both the manufacturing and the development cost. For this reason and despite their typically lower selectivity and worse limits of detection (LOD)^2^, the development of uncoated gas sensors has increased in popularity over the past decade. Indeed, some parameters such as the resonant frequency of a cantilever^6^, the gas sound velocity^7^ and the acoustic attenuation coefficient^8,9^ depend on the gas physical properties. This allowed the development of gas density sensors^10^ and in some cases these principles can be exploited to measure a binary gas mixture concentration^11,12^. Some of these sensors consist of capacitive micromachined ultrasonic transducers (CMUTs)^13^. Typical LODs associated to uncoated sensors go from 3% down to 100 ppm and they are usually considered as non selective. However, in some specific cases, uncoated sensors can show performances close to coated sensors such as a LOD of a few ppm^14^ and even selectivities higher than 10^15^. In order to overcome the problem of selectivity, an alternative consists in discrimination by measuring multiple physical properties of a gas mixture such as mass density and viscosity^16^. In this paper, we propose to measure simultaneously two other gas properties; the acoustic attenuation coefficient, $\alpha$, for frequencies ranging from 1 to 4.5 MHz, and the time of flight, $\tau$, which is the time taken by an acoustic ultrasonic wave to travel from an emitter to a receiver passing through the gas to be characterized. In such a setup, $\alpha$ can be defined as:

1 $$\begin{aligned} \alpha = \frac{1}{d}ln\frac{P_e}{P_r}, \end{aligned}$$

where *d* is the distance between the emitter and the receiver and $P_e$ and $P_r$ are the amplitudes of the acoustic wave at the emission and the reception ends, respectively. This work is the continuation of previous work showing the possibility to use CMUTs to determine the concentration of a binary gas mixture by measuring either $\tau$^17,18^ or $\alpha$^15^ separately and in which the best selectivity obtained was slightly over 10. Although this value is high enough for some applications, it can be considered very low for others. For this reason, a setup capable of measuring both properties, time of flight and attenuation coefficient, is presented in this study. The choice of using CMUTs comes from several advantages over other ultrasonic transducers such as piezoelectric based ultrasonic transducers. These are easier to integrate and present a wider bandwidth, which allows to perform gas measurements over a larger part of the attenuation spectrum^19^. “Results” section shows characterization of the sensor regarding the influence of the temperature and humidity on the sensor and its sensitivity for three mixtures of nitrogen, $\text {N}_{2}$, with either hydrogen, $\text {H}_{2}$, carbon dioxide, $\text {CO}_{2}$, or methane, $\text {CH}_{4}$. The choice of $\text {N}_{2}$ as the main gas is done in order to simplify the modeling and due to its similarity to air in terms of acoustical properties. In “Discussion” section, based on the results, the advantages of measuring simultaneously $\tau$ and $\alpha$, are discussed. Finally, in “Methods” section, details are given about the setup such as the microfabrication of the CMUTs and the signal processing employed for a robust measurement, even in case of spurious reflections and electrical coupling between the emitter and the receiver.

## Results

Details on how both the time of flight, $\tau$, and the shift in attenuation with respect to pure nitrogen at 20 °C, $\Delta \alpha$, are measured can be found in “Methods” section along with detailed information about the setup and the CMUT arrays.

### Temperature and humidity characterization

**Figure 1:**
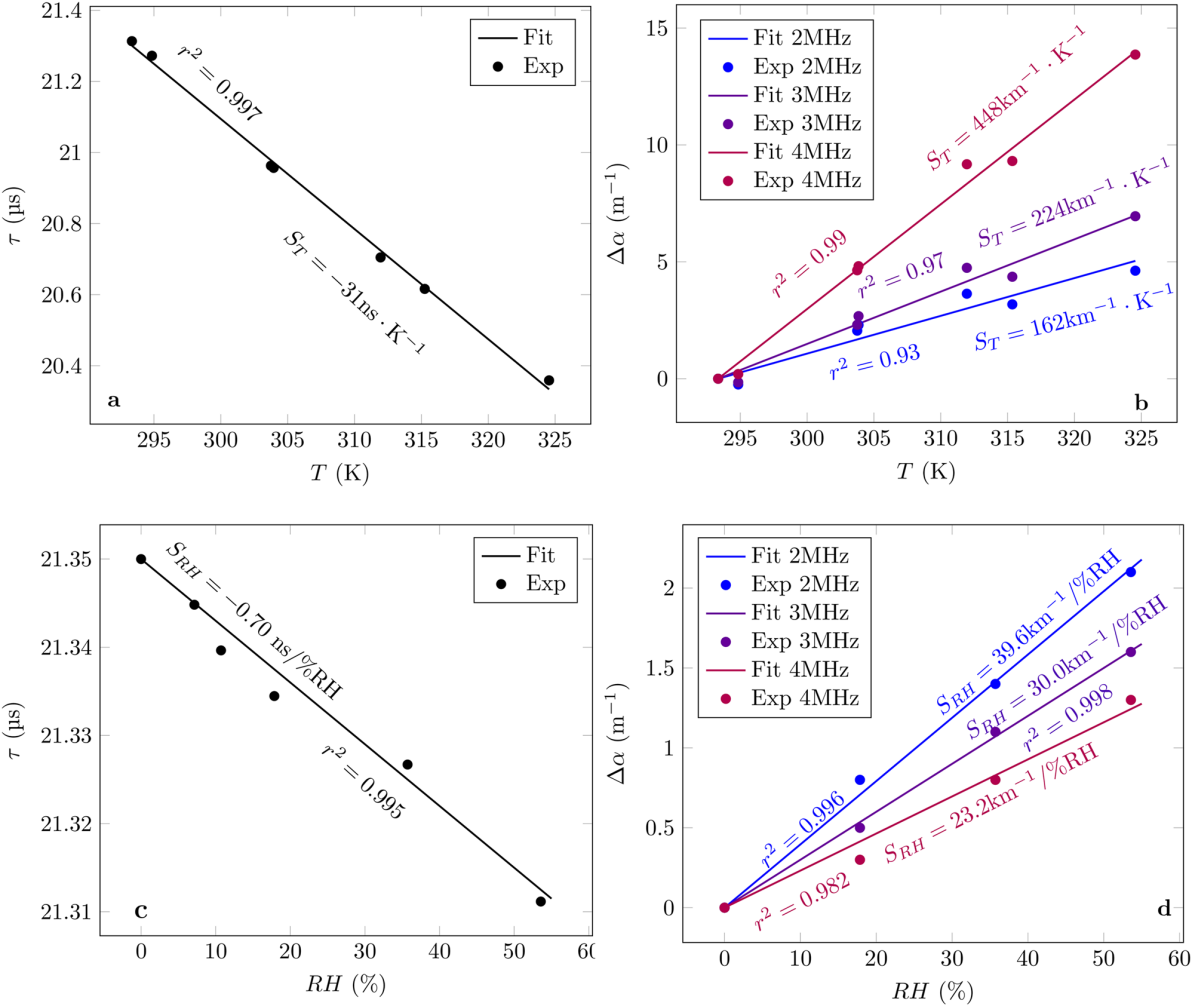
Sensor response to temperature under pure $\text {N}_{2}$: (**a**) time of flight and (**b**) attenuation shift. Sensor response to humidity under pure $\text {N}_{2}$: (**c**) time of flight and (**d**) attenuation shift.

This section seeks to estimate the sensor cross-sensitivity to the temperature, *T*, between 20 and $50 \; ^{\circ }\text {C}$ of both the measurement of $\tau$, $S^{T}_{\tau }$, and of $\Delta \alpha$, $S^{T}_{\alpha }$ as well as the cross-sensitivities to the relative humidity, *RH*, noted $S^{RH}_{\tau }$ and $S^{RH}_{\alpha }$. In order to achieve this, both $\tau$ and $\Delta \alpha$ where measured at different temperatures under pure dry $\text {N}_{2}$ in that temperature range. The resulting characteristics are shown in Fig. 1a,b. Similarly, both quantities, $\tau$ and $\Delta \alpha$, were measured at $20 \; ^{\circ }\text {C}$ with *RH* going from 0% to about 50% (Fig. 1c,d).

In the case of the measurement of $\tau$, the theoretical value can be estimated for an ideal gas by exploiting Newton–Laplace formula for the sound velocity, *v*, of an ideal gas^7^:

2 $$\begin{aligned} v = \sqrt{\frac{\gamma P}{\rho }} =\sqrt{\frac{\gamma R T}{M}}, \end{aligned}$$

where *P* is the atmospheric pressure, $\rho$ the gas mass density, $\gamma$ the heat capacities ratio (considered constant in this range of temperature) and *M* the molar mass of the gas. Differentiating Eq. (2) results in:

3 $$\begin{aligned} \frac{\Delta v}{v} = \frac{1}{2}\frac{\Delta T}{T}, \end{aligned}$$

where $\Delta v$ and $\Delta T$ are small variations of *v* and *T*, respectively. Since $v=d/\tau$,

4 $$\begin{aligned} \frac{\Delta v}{v} = - \frac{\Delta \tau }{ \tau }, \end{aligned}$$

where $\Delta \tau$ represents small variations of $\tau$. Finally, $S^{T}_{\tau }$ is given by:

5 $$\begin{aligned} S^{T}_{\tau } = \frac{\Delta \tau }{\Delta T} = -\frac{1}{2}\frac{\tau }{T} \end{aligned}$$

For pure nitrogen at $20 \; ^{\circ }\text {C}$, $\tau =21.35\,\upmu \text {s}$ (distance $d\approx 7.5 \; \text{mm}$), the theoretical value is $S^{T}_{\tau }=-36 \; \text{ns}/\text{K}$ against the experimental value of $-31\, \text{ns}/\text{K}$. The difference might come from the relatively large temperature variation range or the fact that the sensor and the thermocouple used to measure the temperature are a few centimeters apart inside the oven. However, as it will be shown later, this difference is sufficiently small for the purpose of this article.

In the case of the humidity, one can express the variations of $\tau$ with respect to the molar fraction of vapor water $x_w$ in $\text {N}_{2}$^17^,

6 $$\begin{aligned} \frac{\Delta \tau }{\Delta x_w} = \frac{\tau }{2} \left[ \frac{\rho ^{w}}{\rho ^{N2}}+\frac{c^{w}_{v}}{c^{N2}_v}-\frac{c^{w}_{p}}{c^{N2}_p} -1\right] , \end{aligned}$$

where $\rho ^w = 0.739 \; \text {kg}/\text {m}^{3}$^20^, $c^{w}_p = 1.97 \; \text {kJ/K/kg}$^20^, $c^{w}_v = 1.50 \; \text {kJ/K/kg}$^20^ are the mass density, the isobaric heat capacity and the isochoric heat capacity of water vapor, respectively and $\rho ^{N2}=1.16 \; \text {kg}/\text {m}^{3}$^20^, $c^{N2}_p=1.04$ kJ/K/kg^20^, $c^{N2}_v = 0.743 \; \text {kJ/K/kg}$^20^ the same properties for $\text {N}_{2}$. Since *RH* is the mass of water in the gas with respect to the maximum amount of water that can be diluted in $\text {N}_{2}$, it is given by^20^:

7 $$\begin{aligned} RH = \frac{\rho ^w x_w}{\rho _{max}}, \end{aligned}$$

with

8 $$\begin{aligned} \rho _{max} = \frac{P_sM_w}{RT}, \end{aligned}$$

where $P_s=2310 \; \text {Pa}$^20^ is the saturated water vapor pressure at $20 \; ^{\circ }\text {C}$ and $M_w = 18.02 \; \text {g/mol}$^20^ the molar mass of water. Therefore the sensitivity of $\tau$ to *RH* is:

9 $$\begin{aligned} S^{RH}_{\tau } = \frac{\Delta \tau }{\Delta RH} = \frac{\rho _{max}}{\rho ^w}\frac{\tau }{2} \left[ \frac{\rho ^{w}}{\rho ^{N2}} +\frac{c^{w}_{v}}{c^{N2}_v}-\frac{c^{w}_{p}}{c^{N2}_p}. -1\right] , \end{aligned}$$

Once again the measured value of − 0.70 ns/%*RH* is consistent with the theoretical one of − 0.69 ns/%RH.

In the case of $\Delta \alpha$, due to the complexity of its analytical expression and its dependence on an important number of gas properties, the temperature and humidity influence on the attenuation in this study is purely experimental. From Fig. 1, it can be concluded that, in the range between 2 and 4 MHz, the sensor’s temperature cross-sensitivity increases with the frequency whereas the one of humidity follows the opposite behaviour. Their values will be exploited in the following section.

### Gas characterization

**Figure 2:**
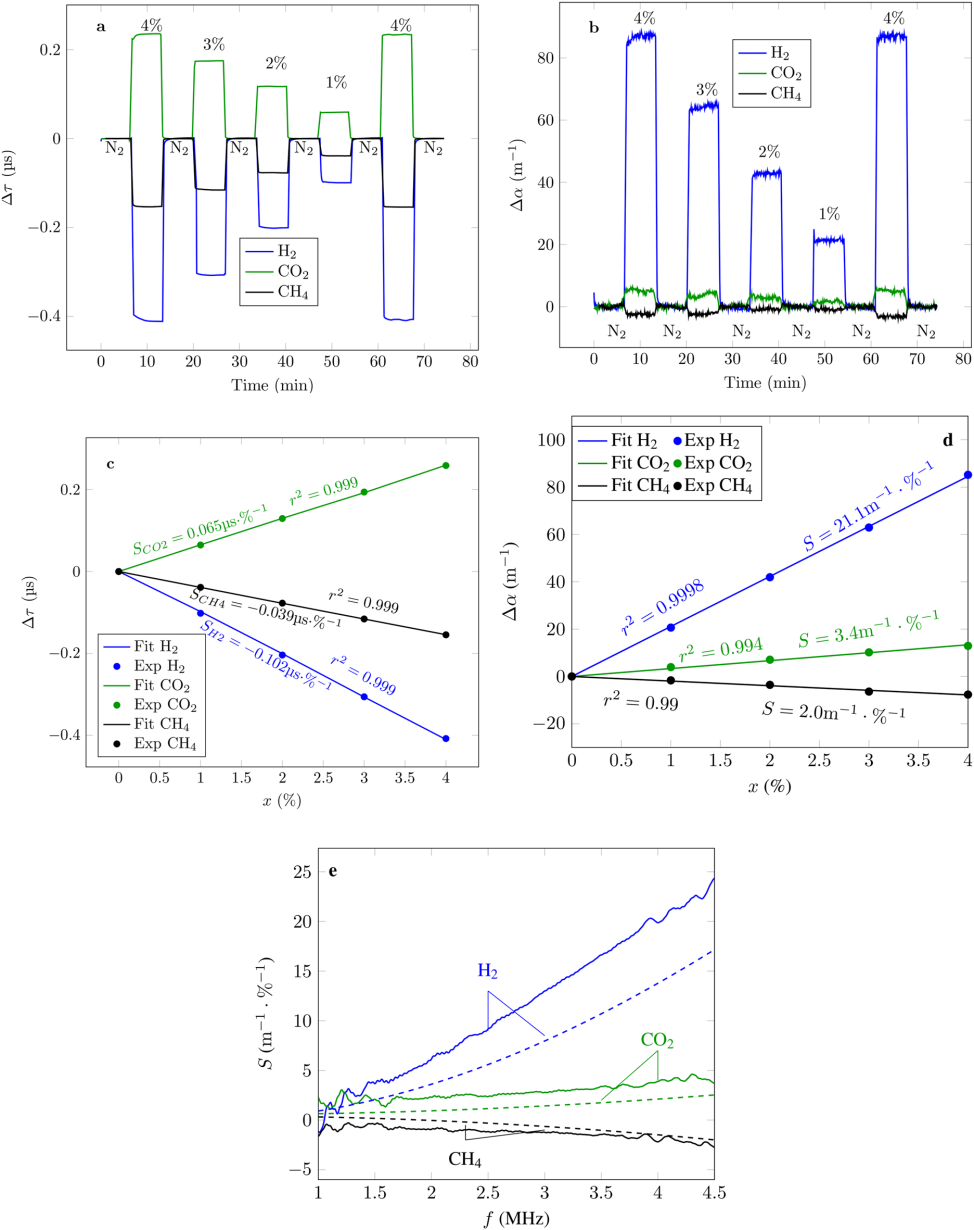
Sensor response to binary mixtures of $\text {N}_{2}$ with either $\text {H}_{2}$, $\text {CO}_{2}$ or $\text {CH}_{4}$. (**a**) Shift in time of flight when alternating the mixture at different concentrations with pure $\text {N}_{2}$. (**b**) Attenuation shift at 4 MHz when alternating the mixture at different concentrations with pure $\text {N}_{2}$. (**c**) Calibration curves from (**a**). (**d**) Calibration curves from (**b**). (**e**) Experimental (solid lines) and theoretical (dashed lines) values of the sensor sensitivity in attenuation shift as a function of the excitation frequency for the three binary gas mixtures.

The temperature and humidity influence on the sensor being known, this section aims to characterize the sensor response to different gas mixtures. For that, the sensor was exposed to sequences where pure $\text {N}_{2}$ is alternated with binary mixtures of $\text {N}_{2}$ with either $\text {H}_{2}$, $\text {CO}_{2}$ or $\text {CH}_{4}$ at different molar concentrations, *x* of the targeted gas. An example of the measurements at 4 MHz is shown in Fig. 2 for both, (a) the shift in time of flight with respect to $\text {N}_{2}$, $\Delta \tau$, and (b) $\Delta \alpha$. From these measurements one can extract the sensitivities through calibration curves, both of $\Delta \tau$ (c) and $\Delta \alpha$ (d) to the targeted gas ($\text {H}_{2}$, $\text {CO}_{2}$ or $\text {CH}_{4}$), $S_{\tau }$ and $S_{\alpha }$, respectively. Although a complete study on the sensor drift (particularly degradation of the mechanical properties of the membrane) is out of the scope of this work, it should be noted that, at constant temperature with dry gases, no detectable drift was observed for several hours of experiment. This emphasizes one of the advantages of uncoated sensors with respect to functionalized sensors.

Since $S_{\alpha }$ depends on the excitation frequency, it was measured for the whole frequency range of 1 MHz up to 4.5 MHz (e) which is limited by the charge amplifier at the lower end and by the strong attenuation above 4.5 MHz. Such frequency range is convenient to prevent typical vibration sources to interfere with the measurements^21^ although it might not be adapted for applications where other ultrasonic sources on the same frequency range as the measurements are present. Similarly to the derivation of the expression of the humidity cross-sensitivity, the expression of $S_{\tau }$ can be obtained^17^:

10 $$\begin{aligned} S_{\tau } = \frac{\Delta \tau }{x} =\frac{\tau }{2} \left[ \frac{\rho ^{G}}{\rho ^{N2}}+\frac{c^{G}_{v}}{c^{N2}_v}-\frac{c^{G}_{p}}{c^{N2}_p} -1\right] , \end{aligned}$$

where $c^{G}_p$ and $c^{G}_v$ are the isobaric and isochoric heat capacities per unit mass of the targeted pure gas, respectively, and $c^{N2}_p$ and $c^{N2}_v$ the ones of pure $\text {N}_{2}$. To derive an exact expression of $S_{\alpha }$ would be unnecessarily complex. Therefore, in this study, a simple model is used from the expression of the attenuation of a pure gas $\alpha _p$^22^:

11 $$\begin{aligned} \alpha _p = \frac{2\pi ^2f^2}{\rho v^3}\left( \frac{4}{3}\eta +\eta _v+\frac{\gamma -1}{c_p}K\right) + \alpha _{vr}, \end{aligned}$$

where $\eta$ is the viscosity of the gas, $\eta _v$ its volume viscosity, *K* its thermal conductivity, $c_p$ its isobaric heat capacity per unit mass and $\alpha _{vr}$ the vibrational relaxation attenuation contribution modeled as^23^:

12 $$\begin{aligned} \alpha _{vr} = \frac{Af}{v}\frac{f/f_{vr}}{1+f^2/f_{vr}^2}, \end{aligned}$$

where *A* is a constant depending on the strength of the relaxation process and $f_{vr}$ the frequency corresponding to the maximum of the normalized attenuation coefficient $\alpha v/f$. When dealing with a binary gas mixture, an additional contribution of the diffusion process $\alpha _\delta$ is needed, especially for molecules with very different molar masses *M* such as $\text {H}_{2}$ and $\text {N}_{2}$^24^:

13 $$\begin{aligned} \alpha _\delta = \frac{2\pi ^2f^2}{\rho v^3}x(1-x)\gamma \delta \left( \frac{\Delta M}{M}+\frac{\gamma -1}{\gamma }a \right) ^2, \end{aligned}$$

where *a* is the thermal diffusion factor of the mixture, $\delta$ its diffusion coefficient and $\Delta M$ is the difference in molar masses between both constituents of the mixture. The shift in attenuation with respect to $\text {N}_{2}$ is then approximated to:

14 $$\begin{aligned} \Delta \alpha = \left[ x\alpha _G + (1-x)\alpha _{N2}+\alpha _\delta \right] -\alpha _{N2}, \end{aligned}$$

where $\alpha _{N2}$ and $\alpha _G$ are the attenuation coefficients in the pure state of $\text {N}_{2}$ and the targeted gas respectively, as given by Eq. (11). Finally, $S_\alpha$ is simply $\Delta \alpha (x=1\%)$.

All the necessary constants to compute both $S_\tau$ and $S_\alpha$ for the three mixtures studied in this article are given in Table 1. From them the theoretical values of $S_\tau$ for the mixtures, $\text {N}_{2}$–$\text {H}_{2}$, $\text {N}_{2}$–$\text {CO}_{2}$ and $\text {N}_{2}$–$\text {CH}_{4}$, are − 100 ns/%, 72 ns/% and − 35 ns/%, respectively. The measurements (Fig. 2c) are consistent with the theory. Additionally, in Fig. 2e, the theoretical values of $S_\alpha$ are displayed in dashed lines along with the measured results. Once again the results are consistent with the theory in terms of sign and the overall form of the variation. In the case of $\text {H}_{2}$, there seems to be some additional losses than the ones predicted by the linear model from Eq. (14). This is due to the beginning of the relaxation attenuation peak of $\text {H}_{2}$, which is already an important and non-linear component of the attenuation^8^. Nevertheless, in a practical application, this can be solved with a calibration mapping $\Delta \alpha$ to the molar fraction of $\text {H}_{2}$ (Fig. 2d).

**Table 1:** Physical properties of gases.

Property	$\text {N}_{2}$	$\text {H}_{2}$	$\text {CO}_{2}$	$\text {CH}_{4}$
$\rho$ (kg/${\text {m}^3}$)	1.16^20^	0.0827^20^	1.82^20^	0.68^20^
$\eta$ ($\upmu \text {Pa}$.s)	17.6^20^	8.8^20^	14.7^20^	11.0^20^
$\eta _v$ ($\upmu \text {Pa}$.s)	12.8^26^	265^26^	5.4^27^	14.5^26^
*K* (W/K/m)	0.024^20^	0.168^20^	0.015^20^	0.030^20^
$c_p$ (kJ/K/kg)	1.04^20^	14.3^20^	0.844^20^	2.22^20^
$c_v$ (kJ/K/kg)	0.743^20^	10.2^20^	0.655^20^	1.70^20^
$\gamma$ (×1)	1.4^20^	1.4^20^	1.3^20^	1.3^20^
*v* (m/s)	349^20^	1306^20^	267^20^	445^20^
$f_{vr}$	0.01 Hz^28^	13 MHz^29^	30 kHz^8^	150 kHz^8^
*A* (×1)	6 × $10^{-4}$^28^	0.56^29^	0.24^8^	6.6 × $10^{-2}$^8^
*M* (g/mol)	28^20^	2^20^	44^20^	16^20^
$\delta$ (${\text {cm}^2}$/s)	NA	0.77^30^	0.15^30^	0.22^30^
*a* (×1)	NA	0.26^31^	0.065^32^	0.070^33^

### Discrimination

**Figure 3:**
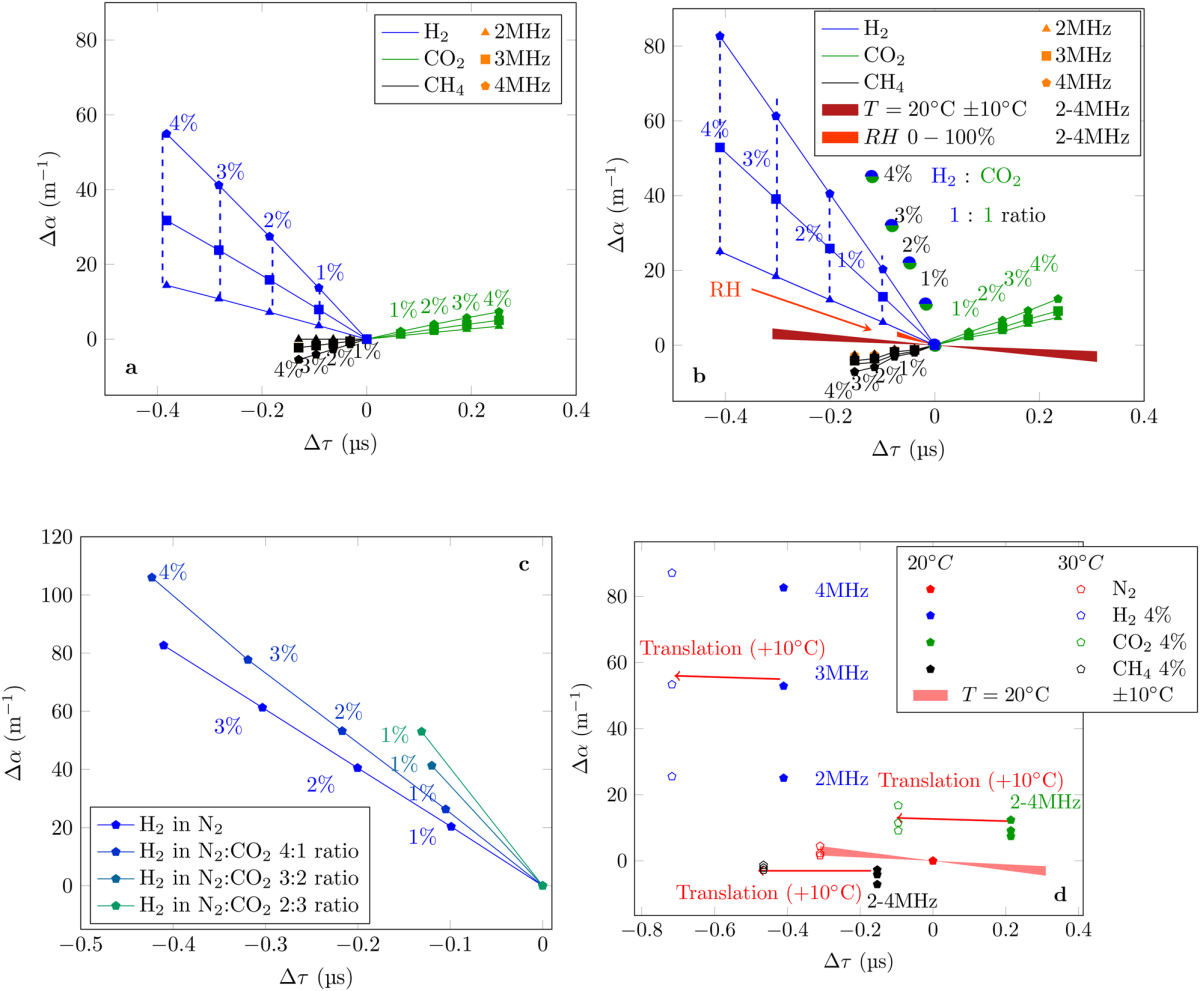
$\Delta \alpha$ as a function of $\Delta \tau$ for different binary gas mixtures. (**a**) Theoretical values. (**b**) Measurements with $\text {N}_{2}$ as reference gas and either pure contaminant ($\text {H}_{2}$, $\text {CO}_{2}$ or $\text {CH}_{4}$) or mixed contaminant ($\text {H}_{2}$ with $\text {CO}_{2}$) at different concentrations. (**c**) Measurements of $\text {H}_{2}$ at different concentrations under different reference gases mixtures of $\text {N}_{2}$/$\text {CO}_{2}$. (**d**) Influence of the temperature on the discrimination resulting in a translation effect.

So far, the measurements of $\Delta \tau$ and $\Delta \alpha$ have been displayed separately. However they are always measured simultaneously. Therefore, one can exploit this in order to identify the different mixtures. Indeed, as it is shown in Fig. 3a, in theory (Eqs. 10 and 14), when plot $\Delta \alpha$ against $\Delta \tau$, the three different types of mixtures are placed in different parts of the plane. Additionally, since in absolute value, $S_\alpha$ increases with the frequency, it is easier to distinguish between them at higher frequencies (4 MHz rather than at 2 MHz). Figure 3b shows the corresponding measurements resulted from the previous characterization as well as the influence of the temperature and humidity, taking into account the cross-sensitivities measured in “Temperature and humidity characterization” section. In the case of the humidity the values above 50% RH were extrapolated to estimate the influence up to 100% RH assuming that the cross-sensitivities of both $\tau$ and $\Delta \alpha$ remain constant. Overall the humidity influence remains weak compared to the one of the temperature. In order to illustrate what would happen with a contaminant which is not a pure gas but a mixture of two gases with a fixed constitution (1:1 ratio in this case), measurements of a mixture of $\text {H}_{2}$ and $\text {CO}_{2}$ at 4 MHz at different concentrations (1% of the mixture contains 0.5% of each gas) are shown (Fig. 3b). As one would expect, the points fall in between the curves at 4 MHz of each of the constituents. In Fig. 3c, preliminary results at 4 MHz of how the measurements using $\text {N}_{2}$ as reference gas are modified by using a different reference such as a mixture of $\text {N}_{2}$ and $\text {CO}_{2}$ are shown. In fact, if the reference gas is changed, the sensitivities are modified and consequently the slopes of the curves in the plane ($\Delta \alpha$, $\Delta \tau$) change as well. However, if the reference gas is known, this can be taken into account during the calibration step (Fig. 2c, d). Finally, additional measurements detecting 4% of each of the three mixtures ($\text {N}_{2}$ with either $\text {H}_{2}$, $\text {CO}_{2}$ or $\text {CH}_{4}$) at 30 °C and at 1, 2 and 3 MHz were performed and displayed with the corresponding measurements at 20 °C in Fig. 3d. From them it can be observed that the main influence of the temperature is an horizontal displacement (shift in $\tau$) with a relatively small displacement along the axis of $\Delta \alpha$.

## Discussion

The CMUT ultrasonic gas sensor presented in this paper allows to measure simultaneously both the time of flight and the attenuation coefficient in a binary gas mixture. This allows to discriminate, at a fixed temperature and fixed humidity, between the proposed binary mixtures of $\text {N}_{2}$ with either $\text {H}_{2}$, $\text {CO}_{2}$ or $\text {CH}_{4}$. Remarkably, a change in the sensor output due to the temperature or humidity can be distinguished by a small change in the attenuation coefficient and a large shift in the time of flight especially for the temperature. This means that, to some extent the sensor presents also temperature or humidity sensor capabilities that can help for numerical compensation. This can be exploited even further by the fact that a slight increase in *T* from 20 to 30 °C or change in relative humidity results mainly in a substantial decrease in $\tau$ with a small shift in $\Delta \alpha$. This means that if a slight loss in accuracy (due to the minor distortion in attenuation caused by either the temperature or the humidity) can be afforded, whenever a change in *T* or *RH* is detected (delimited by the dark and light red zones in Fig. 3b), the sensor can perform a self recalibration compensating such effect. Additionally, after the mixture is identified, the concentration can be determined with calibration curves. Concretely, the simultaneous measurement of time of flight and attenuation coefficient with CMUTs results in a robust three binary gas mixtures sensor with built in temperature or humidity sensor capabilities and long term stability. Some potential applications are monitoring of refueling stations, vehicles and nuclear waste storage facilities. Preliminary results in the case of more complex mixture have been presented as well which showed that the calibration step has to be adapted to the application.

## Methods

### CMUT details

**Figure 4:**
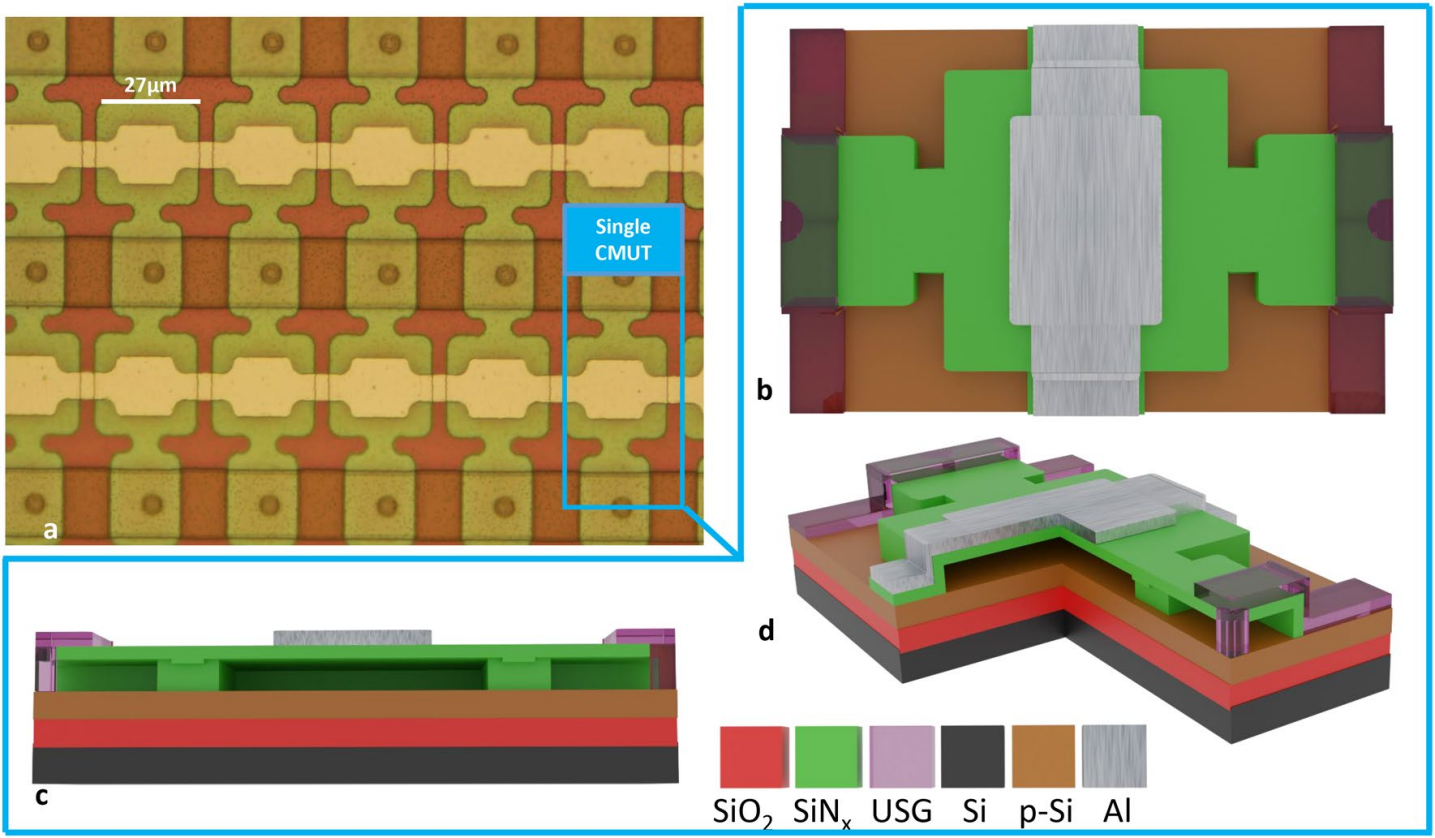
Sensor used in this study. (**a**) Optical microscope image showing part of the CMUT array. (**b**)–(**d**) 3D sketch showing the top view, a transversal cut and a partial cut respectively, where USG stands for Undoped Silicon Glass. Sketch not to scale for visualization purposes.

An optical microscope picture of the CMUT array used in this study is shown in Fig. 4 along with 3D schematics from different perspectives of single CMUT. The microfabrication protocol used is similar to the one presented in^25^. Their geometrical characteristics along with polarization and excitation details are given in Table 2.

**Table 2:** CMUTs characteristics.

Membrane size (μm × μm)	27 × 27
Gap (nm)	250
Collapse voltage (V)	98
Reception DC voltage (V)	70
Emission DC voltage (V)	50
Emission AC voltage peak to peak (V)	40
Resonant frequency (MHz)	9.6
Quality factor (×1)	30

### Setup

**Figure 5:**
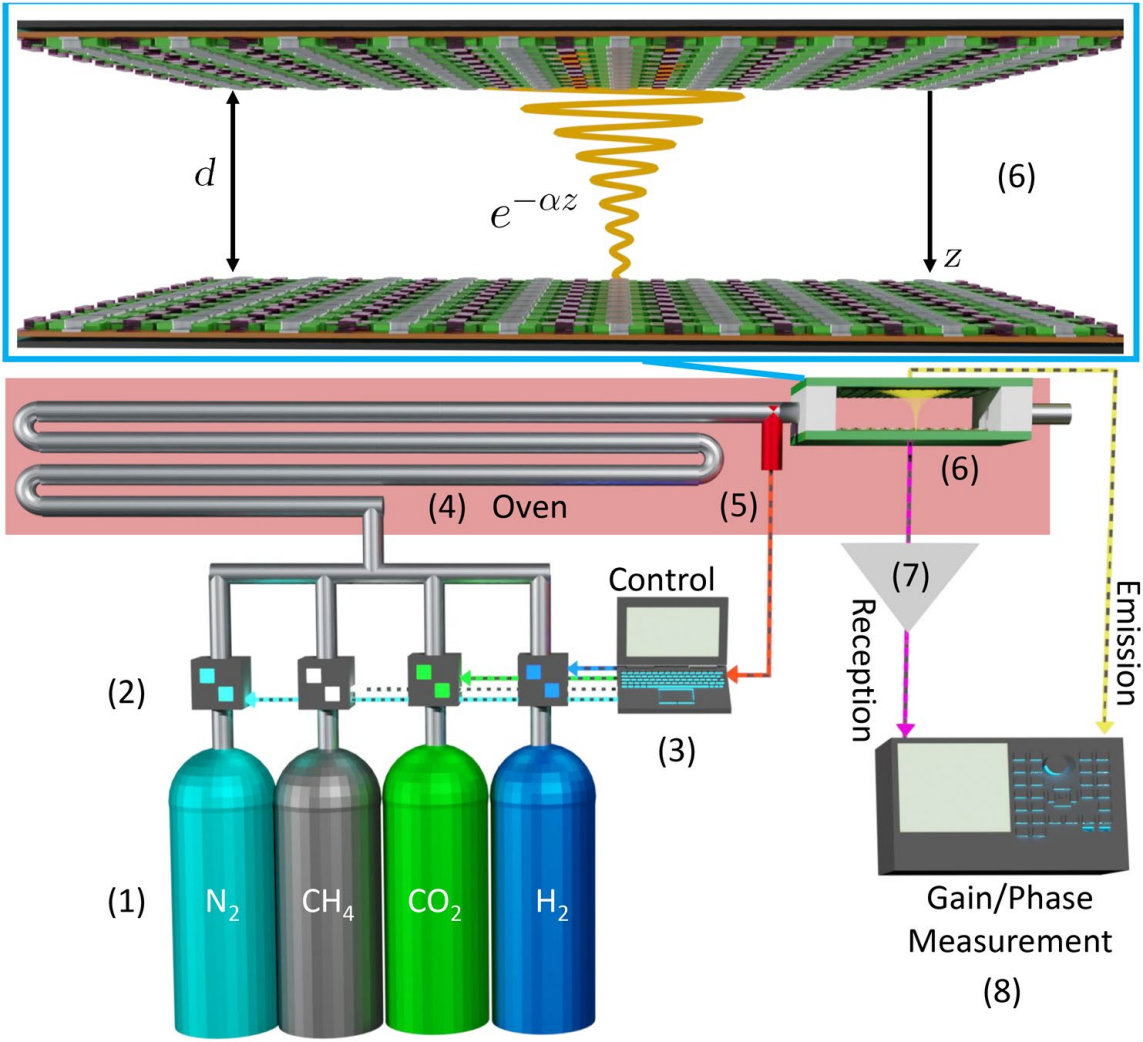
Complete setup schematics. (1) Gas bottles, (2) flowmeters, (3) flow and temperature control computer, (4) oven, (5) thermocouple, (6) measurement cell with zoom at the top of the image, (7) charge amplifier, (8) gain/phase network analyzer.

The setup to measure both the shift in attenuation, $\Delta \alpha$, and the sound velocity, *v*, for different gases at different concentrations is shown in Fig. 5. The gases from industrial grade bottles (1) are mixed by a system of flowmeters (2) controlled by a computer (3) at the desired concentrations. The mixture passes through an oven (4) where the desired temperature is set and verified by a thermocouple (5). The gas enters the measurement cell (6) where a continuous ultrasonic sine wave is sent from an emitter CMUT array to a receiver CMUT array after travelling a distance *d*. Thus, the wave arrives attenuated exponentially with *d*. Additionally its phase is shifted with respect to the emission by an amount proportional to the time of flight, $\tau =d/v$, required to go through the measuring cell. The receiver is connected to a charge amplifier (7) in order to improve the signal to noise ratio before being fed along with the emission signal to a gain/phase network analyzer (8). Finally, for the humidity measurements, a vapor generator PUL110 was used to humidify $\text {N}_{2}$. It should be noted that although the measuring setup presented is rather bulky, more compact components such as the network analyzer can be built^34^.

### No reflections model

Far from the resonance of the CMUTs, on the side of lower frequencies, one can assume that their electromechanical transfer function is a constant. Thus, using the complex formalism, the pressure wave *p* can be considered of the form:

15 $$\begin{aligned} p(t,z) = P_{00}e^{-\alpha z}e^{j(\omega t -kz)}, \end{aligned}$$

where $P_{00}$ is the amplitude of the wave at $t=0$ on the emission end, $\omega$ the excitation angular frequency, $j=\sqrt{-1}$, *t* the time, *z* the coordinate orthogonal to the CMUT array (Fig. 5), $\alpha$ the attenuation coefficient of the gas and *k* the wave number which, in the case of a plane wave, is linked to the gas sound velocity *v* as follows:

16 $$\begin{aligned} k = \frac{\omega }{v}. \end{aligned}$$

Since the wave is continuous, at any given time $t_0\ge \tau$, the value of *p* at the receiving end ($z=d$) is:

17 $$\begin{aligned} p(t_0,d)=P_{00}e^{-\alpha d}e^{j\omega t_0}e^{-j\omega d/v}. \end{aligned}$$

Thus, its phase is shifted by a value of $-\omega \tau$ ($\tau =d/v$) with respect to the value of *p* at the emission end at the same time $t_0$:

18 $$\begin{aligned} p(t_0,0)=P_{00}e^{j\omega t_0}. \end{aligned}$$

The transfer function due only to the setup, $H_0$, independent of the gas can be defined as:

19 $$\begin{aligned} H_0 = |H_0|e^{j\phi _0}, \end{aligned}$$

where $\phi _0$ is a constant and accounts for the shifts due to the setup and $|H_0|$ is the modulus of $H_0$, which depends only on the angular frequency. Therefore the total phase shift $\phi$ is:

20 $$\begin{aligned} \phi = -\omega \tau +\phi _0, \end{aligned}$$

As a consequence $\tau$ is simply the slope of the curve $\phi (\omega )$. Additionally, the change in amplitude is simply $e^{-\alpha d}$. Hence the total gain |*H*| is given by:

21 $$\begin{aligned} |H| = |H_{0}(j\omega )|e^{-\alpha d}, \end{aligned}$$

In order to overcome the need of knowing $|H_{0}|$, a calibration step was performed to measure |*H*| under $\text {N}_{2}$, $|H|_{N2}$. Indeed, the shift in attenuation with respect to $\text {N}_{2}$, $\Delta \alpha$, is given by:

22 $$\begin{aligned} \Delta \alpha = \frac{1}{d}ln\frac{|H|_{N2}}{|H|}, \end{aligned}$$

### Dealing with multiple reflections

In order to measure a high signal to noise ratio, especially for higher frequencies, it is possible to increase the excitation amplitude. However, this will cause reflections that will interfere with the main emission, particularly for lower frequencies, where the attenuation is smaller. Therefore, they will modify both $\phi$ and |*H*|. This section seeks to understand the extent of this effect and, particularly, how to retrieve information about both $\alpha$ and $\tau$. Assuming normal incidence and a reflection coefficient of *r* identical for both CMUT arrays, at a given position *z* and time *t*, the acoustic wave in the cell is the superposition of a wave travelling forward $p_f$ (towards increasing *z*) and one travelling backwards $p_b$ (towards decreasing *z*). The former is the sum of all the reflections that happen an even number of times (0 included) between the emission time and the measurement time i.e.:

23 $$\begin{aligned} p_{f,n} = P_{00}e^{j\omega t}\sum _{i=0}^{n} r^{2i}e^{-(jk+\alpha )(2id+z)}, \end{aligned}$$

where *n* is the echo order (initial 0). Similarly, $p_b$ is the sum of the waves that have been reflected an odd number of times.

24 $$\begin{aligned} p_{b,n} = P_{00}e^{j\omega t}\sum _{i=1}^{n} r^{2i-1}e^{-(jk+\alpha )(2id-z)}. \end{aligned}$$

Notice that the term of order 0 is not to be accounted since the emission happens only towards the increasing *z*. Both $p_f$ and $p_b$ are geometric sums of same quotient *q* given by:

25 $$\begin{aligned} q = r^2e^{-2d(jk+\alpha )}. \end{aligned}$$

Since $|q|<1$, $p_f$ and $p_b$ are simply given by:

26 $$\begin{aligned} p_{f,n}= & {} p_{f,0}\frac{1-q^{n+1}}{1-q}, \end{aligned}$$

27 $$\begin{aligned} p_{b,n}= & {} u_{0}\left( \frac{1-q^{n+1}}{1-q} -1\right) , \end{aligned}$$

where

28 $$\begin{aligned} p_{f,0}= & {} P_{00}e^{j(\omega t -kz) -\alpha z}\end{aligned}$$

29 $$\begin{aligned} u_{0}= & {} \frac{1}{r}P_{00}e^{j(\omega t + kz) +\alpha z}. \end{aligned}$$

The total acoustic signal is finally

30 $$\begin{aligned} p_n = p_{f,n}+p_{b,n}. \end{aligned}$$

One can verify that, the case $n=0$ corresponds exactly to the model presented in “No reflections model” section. In permanent regime, one can consider the number of echoes to be infinite, resulting in the final acoustic signal $p_\infty$:

31 $$\begin{aligned} p_\infty (z) = \frac{p_{f,0}(z)+qu_{0}(z)}{1-q}. \end{aligned}$$

The total transfer function *H* is then given by:

32 $$\begin{aligned} H = -H_0\frac{p_\infty (z=d)}{P_{00}} \end{aligned}$$

which equals:

33 $$\begin{aligned} H = -H_0\frac{(r+1)e^{-j\omega \tau -\alpha d}}{(re^{-j\omega \tau -\alpha d}-1)(re^{-j\omega \tau -\alpha d}+1)} \end{aligned}$$

It should be noticed once again that without any reflections ($r=0$) $H(j\omega )$ is equal to the transfer function with a single peak:

34 $$\begin{aligned} H_{n=0} = H_0e^{-j\omega \tau -\alpha d}. \end{aligned}$$

In order to extract information about $\alpha$ some additional calculations are needed. Indeed, by using the fact that for $|y|<1$:

35 $$\begin{aligned} \frac{1}{1-y} = \sum _{n=0}^{\infty }y^n, \end{aligned}$$

and some basic series manipulations Eq. (33) can be rewritten as:

36 $$\begin{aligned} H = \sum _{n=1}^{\infty } P_ne^{-j\omega (2 n-1) \tau }, \end{aligned}$$

where

37 $$\begin{aligned} P_{n}=H_{0}(r+1)r^{2(n-1)}e^{-(2n-1)\alpha d}. \end{aligned}$$

It is important to point out that all the $P_n$ are independent of $\tau$. From this form it can be seen that *H* is the sum of complex exponentials modulated by the functions $P_n$. Applying the inverse Fourier transform to *H* (noted ${{\hat{H}}}$) defined as:

38 $$\begin{aligned} {{\hat{H}}}=\int ^{\infty }_{-\infty } H(f)e^{2 \pi jf t}df, \end{aligned}$$

will result in:

39 $$\begin{aligned} {{\hat{H}}}(t)= \sum _{n=1}^{\infty } \hat{P_n}(t-(2n-1)\tau ), \end{aligned}$$

where $\hat{P_n}$ is the inverse Fourier transform of $P_n$. Therefore, ${{\hat{H}}}$ is the sum of peaks, where the envelope is the shifted inverse Fourier transform of $P_n$. In particular, the amplitude of the second peak is given by:

40 $$\begin{aligned} P_1 = H_{0}(r+1)e^{-\alpha d}. \end{aligned}$$

**Figure 6:**
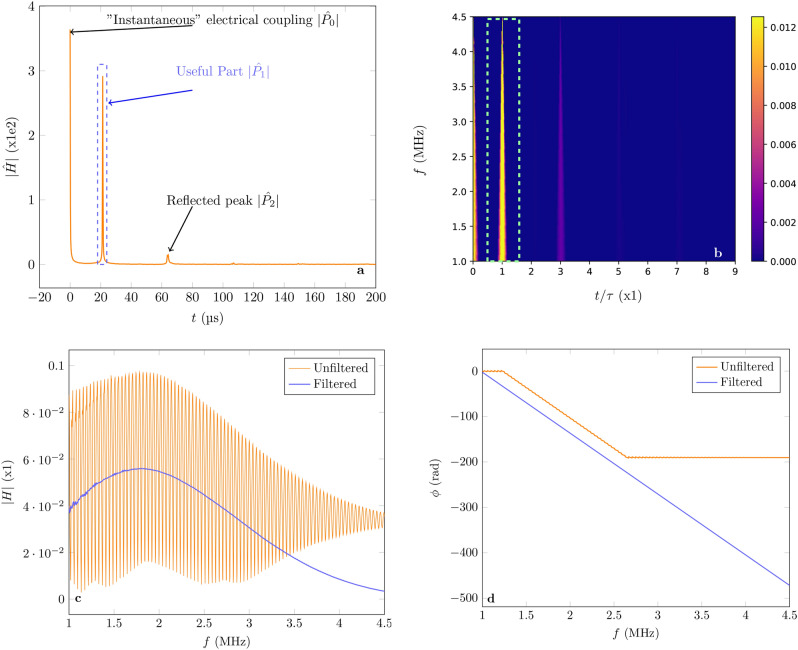
(**a**) Measurement of $|{{\hat{H}}}|$ as a function of the delay with respect to the emission. (**b**) Its wavelet transform diagram. (**c**) and (**d**) Comparison between the transfer function for multiple reflections (unfiltered) and after the main echo is extracted (filtered) for the gain and phase, respectively.

Therefore, by isolating $|P_1|$ it is possible to apply the same formula from the single reflection case (Eq. 22) to retrieve $\Delta \alpha$. Figure 6a shows the measured result of Eq. (38) under pure $\text {N}_{2}$ as a function of the delay with respect to the emission, *t*, which is consistent with Eq. (39). It should be noted that, apart from the main peak $\hat{P_1}$ (at $t=\tau$) and the reflected echo $\hat{P_2}$ (at $t=3\tau$) there is an additional peak at $t=0$. This is due to an instantaneous electrical coupling between the emitter and the receiver resulting from the high voltages applied^17^. However, since the objective is to isolate $P_1$, the coupling won’t influence the final result. Figure 6b shows the wavelet transform of the measurement from Fig. 6a and shows that the main peak contains more spectral information, since the highest frequencies are no longer present in the reflected echo. This supports the fact that in order to have information about the attenuation at the highest frequencies it is useful, and sometimes necessary, to accept the presence of reflections of the lower frequency components constituting the acoustic wave. However, this perturbs strongly *H*. This can be appreciated in Fig. 6c,d, which show the comparison between the measured transfer function (both with multiple reflections and electrical coupling) and the result of the data processing after retrieving only the contribution of $P_1$ by filtering out components of ${{\hat{H}}}$, which are outside of the range $t\in [15,30] \; \upmu \text {s}$. This numerical criterion should be modified according to the application (*v* and *d*). In our case, $\tau$ is not expected to vary more than $1 \; \upmu \text {s}$. It should be noted that, after filtering, |*H*| is smooth as one would expect from the case without reflections. Moreover, the unwrapped (2$\pi$-modulo removed) phase after filtering is consistent with the model of a single peak as shown in “Results” section its slope corresponds to $-2\pi \tau$.
